# Exploratory study of nanoparticle interaction with intraorally formed dental biofilms

**DOI:** 10.1186/s12903-025-06703-x

**Published:** 2025-08-22

**Authors:** Anton Schestakow, Maria Riegelmann, Matthias Hannig

**Affiliations:** Clinic of Operative Dentistry, Periodontology and Preventive Dentistry, University Hospital, Saarland University, Building 73, 66421 Homburg/Saar, Germany

**Keywords:** Biofilms, Nanoparticles, Electron microscopy, Dental enamel, Dental pellicle, Preventive dentistry

## Abstract

**Background:**

The development of nanoparticles offers promising potential for improving biofilm management; however, the biofilm itself acts as a diffusion barrier, limiting effective treatment. This study aimed to investigate the adsorption and diffusion of nanoparticles in an intraorally formed biofilm.

**Methods:**

Bovine enamel specimens (*n* = 24) were mounted on customized maxillary splints and worn intraorally by two subjects for 24 h to allow biofilm formation. Specimens not exposed to the oral cavity served as controls (*n* = 12). Ex vivo, 20 nm gold nanoparticles with a low-charge polymer outer layer were applied to the biofilm for 10 to 30 min, followed by either a single wash, 20 washes with water, or 24 h of water storage. The outer surface and basal layer of the biofilm were analysed using scanning electron microscopy, while cross-sections were examined using transmission electron microscopy.

**Results:**

After 24 h of intraoral exposure, enamel was covered by a globular-structured pellicle with bacterial adhesion and occasional biofilm formation, more pronounced in subject 2. Both facilitated nanoparticle adsorption, which increased with exposure time and remained detectable after 20 washes. In subject 2, distinctly more nanoparticles persisted after 24 h of water storage. Transmission electron microscopy confirmed outer surface retention without penetration into deeper biofilm layers.

**Conclusions:**

The diffusion of 20 nm nanoparticles in dental biofilms appears limited, leaving open questions regarding the optimal nanoparticle size for effective biofilm management and their toxicological implications.

## Introduction

Dental caries and periodontal diseases are among the most prevalent biofilm-associated diseases, affecting billions of people worldwide [1]. These multifactorial diseases are caused by bacterial colonization of dental surfaces, where bacteria form biofilms, multicellular communities embedded in an extracellular matrix. Biofilms are inherently resistant to antibacterial substances due to their reduced permeability and ability to develop resistance mechanisms. Consequently, the primary treatment for caries and periodontitis relies on mechanical biofilm removal, supplemented by the use of fluorides and antiseptics [2, 3]. However, mechanical biofilm removal has its limitations, particularly in interdental spaces, which are the predilection sites for caries and periodontitis [4]. While fluorides can prevent caries and antiseptics can inhibit biofilm formation, the biofilm itself acts as a diffusion barrier for many substances. The high global prevalence of these diseases suggest that current treatment strategies are not sufficient enough [5], underscoring the need to develop alternative or complementary approaches.

In the era of nanotechnology, nanoparticles offer potential to address the challenges posed by biofilm-associated diseases. These materials, ranging in size from 1 to 100 nm, exhibit unique and versatile properties that make them promising in biomedical applications. Some nanoparticles, such as metal-based nanoparticles, possess intrinsic antibacterial properties, while others function as carriers for targeted drug delivery [6]. The interaction of nanoparticles with biofilms follows three sequential phases, which is supported by the high specific surface area of nanoparticles. First, nanoparticles are transported to the biofilm, for instance, by bulk flow through a mouth rinsing solution. Second, nanoparticles adsorb onto the biofilm outer surface. Third, they diffuse within the biofilm through water compartments and channels via passive diffusion [3], which is influenced by several factors: the pore size and physicochemical properties of the biofilm on one hand, and the composition, size, and surface charge of the nanoparticles on the other. Notably, the physicochemical properties of nanoparticles can be modified to enhance their performance [6]. For example, smaller nanoparticles with a positive charge are more effective at penetrating the negatively charged biofilm [3, 7].

Effective dental biofilm control requires anti-biofilm agents to adsorb strongly to the biofilm or dental surface to achieve long-lasting efficacy and to penetrate into the biofilm to exert antibacterial effects by directly interacting with bacteria across all biofilm layers [8]. Dental biofilm formation is a dynamic process that begins with the adsorption of salivary proteins onto dental surfaces, leading to the formation of a pellicle. This pellicle acts as a substrate for bacterial adhesion [9], where bacteria produce an extracellular matrix and ultimately develop a biofilm composed of hundreds of bacterial species. Despite this complexity, most studies have relied on in vitro mono- or multispecies biofilm models grown on non-dental surfaces, which do not accurately replicate the dynamic nature of dental biofilm formation [10, 11].

Therefore, the aim of this study was to investigate the interaction of nanoparticles in dental biofilms formed in situ on enamel specimens within the oral cavity of subjects with varying biofilm formation rates. Gold-core nanoparticles with a low-charge coating were used to enable identification via electron microscopy and to minimize the influence of surface charge. The null hypothesis was that nanoparticles do not exhibit strong adsorption to the biofilm and do not further penetrate the biofilm during extended water storage.

## Materials and methods

### Specimens

The enamel specimens were prepared from the labial surfaces of lower incisors from 2-year-old cattle, obtained from a local slaughterhouse (Schlachthof Emil Faerber, Zweibruecken, Germany). The teeth and all intermediates were stored in 0.1% thymol at 4 °C. After removing the roots and lingual surfaces with a circular saw, the labial surfaces were cut into smaller pieces and wet-ground to create rectangular specimens (5 × 5 × 1.5 mm). The enamel surfaces were polished to 4000-grit using silicon carbide abrasive paper. To remove the smear layer, specimens were treated with 3% sodium hypochlorite for 3 min, rinsed in water (4 × 5 min), and placed in an ultrasonic bath for 5 min. Disinfection was carried out in 70% isopropyl alcohol for 15 min, followed by rehydration in water for 24 h.

### Subjects

Two subjects participated in the study: subject 1 (female; 26 years) with a low biofilm formation rate and subject 2 (male; 41 years) with a high biofilm formation rate, as determined by preliminary 24-h intraoral biofilm formation tests and analyses using polarized light microscopy and scanning electron microscopy. Both subjects were caries-free, in good general health, and reported no use of medications, alcohol, or nicotine. Ethical approval was obtained from the Medical Association of Saarland (238/03, 2016). The study design is in accordance with the Declaration of Helsinki. Signed informed consent forms were obtained from all of the participants in the study.

### Nanoparticles

**Fig. 1:**
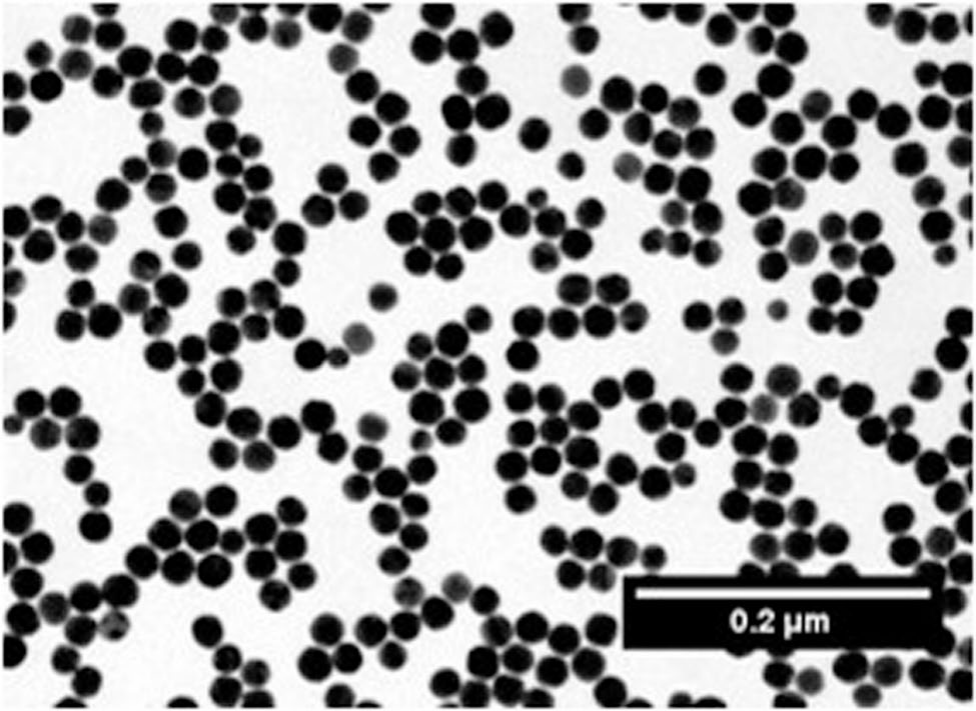
TEM image of colloidal gold nanoparticles

Gold nanoparticles (In Vitro Fluorescent Spherical Gold NPs 10 nm, Nanopartz, Loveland, CO, USA) were used, provided as a colloid in phosphate buffered saline at a concentration of 3.5 mg/mL. The nanoparticles consisted of a gold core, a SiO_2_ polymer spacer, and an outer layer of methyl polymer containing fluorophores. According to the manufacturer, dynamic light scattering measurements indicated a zeta potential of + 17 mV and a polydispersity index of 0.15, suggesting moderate colloidal stability and a relatively narrow size distribution. A fresh stock solution was prepared by diluting the nanoparticles 1:10 with distilled water (Aqua B. Braun, B. Braun Melsungen, Melsungen, Germany) after 5 min of sonication. Transmission electron microscopy (TEM) (Tecnai 12, FEI Company, Eindhoven, Netherlands) showed highly electron-dense spheres with an average diameter of 20 nm, which appeared homogeneously distributed (Fig. 1).

### Biofilm formation and treatment

**Fig. 2:**
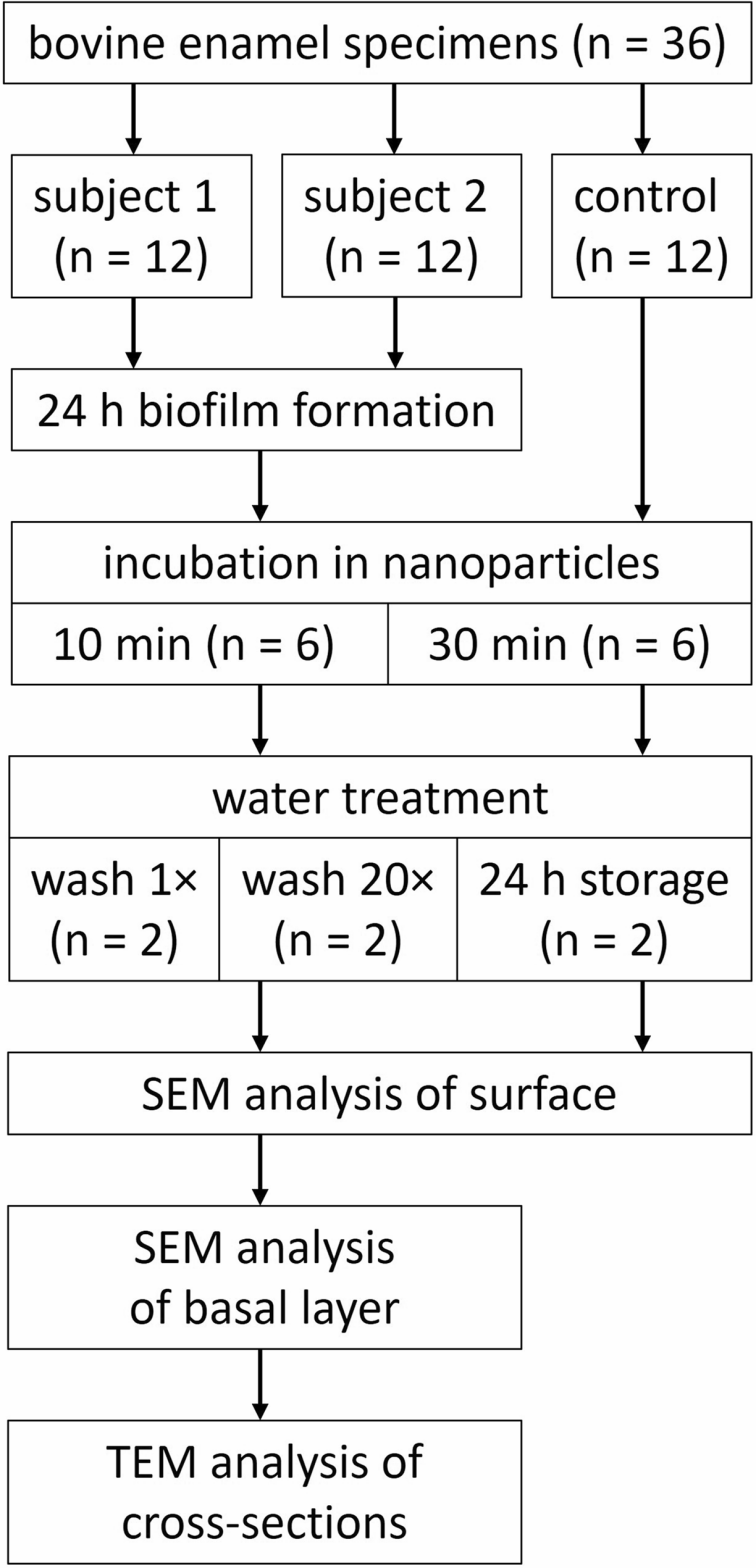
Flow chart

Specimens were fixed to the buccal sites in the region of the maxillary premolars and first molars on customized splints made of methacrylate (Duran, Scheu-Dental, Iserlohn, Germany) using silicone impression material (President Light Body, Coltene/Whaledent, Langenau, Germany). The splints were worn intraorally for 24 h by both subjects to allow biofilm formation, while specimens without intraoral exposure served as controls (Fig. 2). The splints were worn continuously and were only temporarily stored in a moist chamber during food intake. Oral hygiene was omitted during this period. After 24 h, the biofilms were fixed in 2% glutaraldehyde in 0.1 M cacodylate buffer for 1 h, washed with 0.1 M cacodylate buffer (5 × 10 min), post-fixed with 2% osmium tetroxide for 1 h, and washed again in 0.1 M cacodylate buffer (5 × 10 min). The specimens were incubated in 50 µl of nanoparticles for 10–30 min. After incubation, they were washed once or 20 times with distilled water, or stored in distilled water for 24 h. Finally, the specimens were air-dried for 24 h.

### SEM analysis

Specimens were sputter-coated with carbon and analysed using scanning electron microscopy (SEM) (XL 30 ESEM FEG, FEI Company, Eindhoven, Netherlands) at magnifications of up to 50,000×. A mixed imaging mode was used, consisting of 30% secondary electrons (SE) and 70% backscattered electrons (BSE), to visualize organic structures and gold nanoparticles, respectively. Four randomly captured images per specimen, each covering 100 μm² and representing approximately 0.0016% of the total specimen surface, were analysed at 10,000× magnification. The number of gold nanoparticles was automatically counted using MATLAB 8.6 (The MathWorks, Natick, MA, USA). The specimens of subject 1 and 2 were then embedded in epoxy resin (Araldite Resin CY212, Agar Scientific, Stansted, UK). The dentin was removed using a wet grinding machine and silicon carbide abrasive paper, while the enamel was removed by treatment with 1 M HCl for 2 h. After sputter-coating with carbon, the basal layer of the biofilm was examined at up to 50,000× magnifications.

### TEM analysis

After the SEM analyses of the embedded specimens from subjects 1 and 2, the specimens were re-embedded in epoxy resin. Ultrathin sections were cut using an ultramicrotome (Leica EM UC7, Leica Microsystems, Wetzlar, Germany) equipped with a diamond knife (Diamond Knives Ultra 45, DiATOME, Hatfield, PA, USA). The sections were mounted on pioloform-coated grids, contrasted with uranyl acetate and lead citrate, and 15 to 59 TEM-images were captured at magnifications of up to 150,000×.

## Results

### SEM analysis

**Fig. 3:**
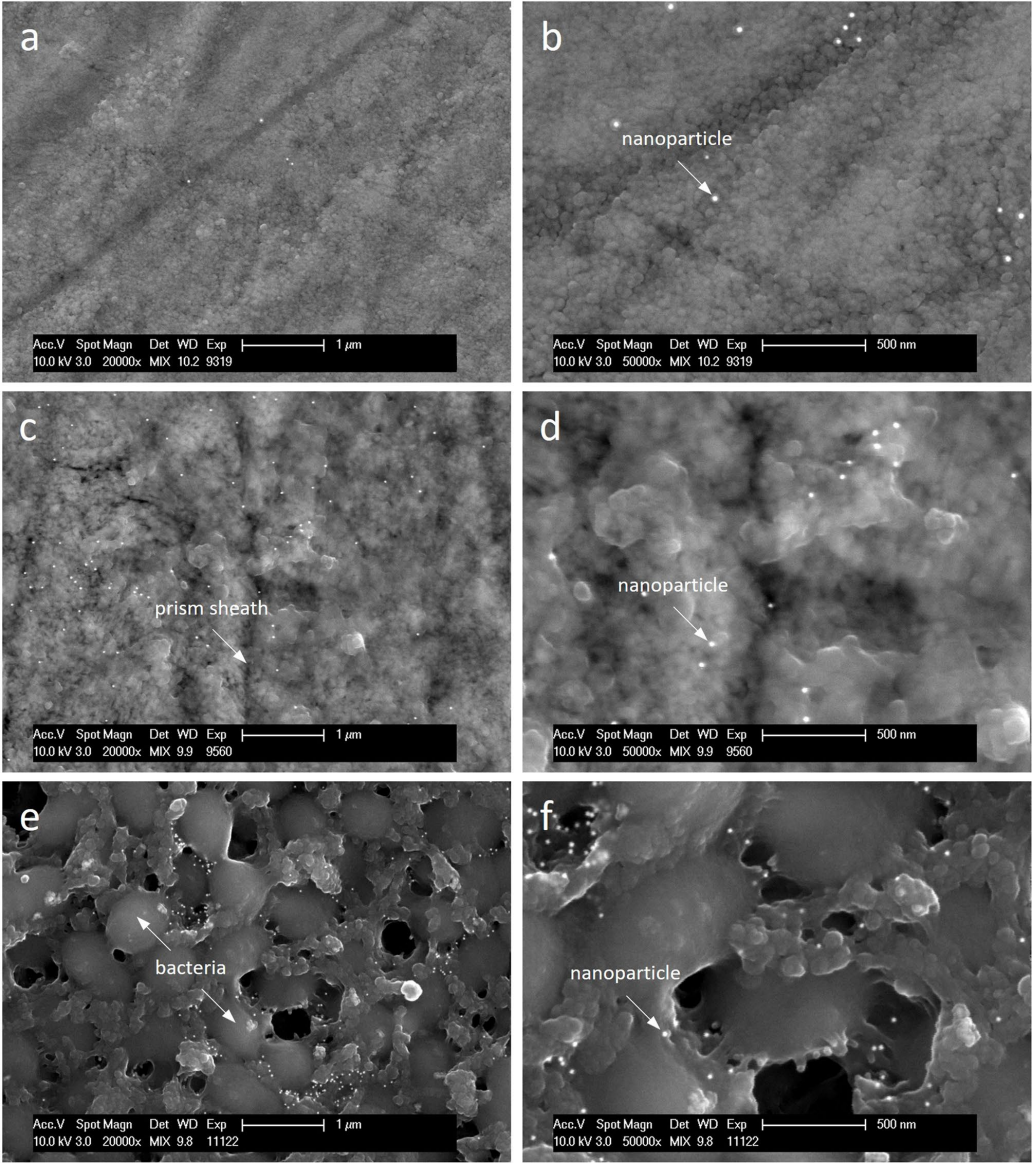
SEM images of enamel specimen incubated with gold nanoparticles for 10 min either directly (**a**, **b**) or after intraoral exposure in subjects 1 (**c**, **d**) and 2 (**e**, **f**), followed by 20 washes with water. After intraoral exposure, the prismatic enamel was covered by a globular pellicle layer with adhered bacteria. Both the pellicle and biofilm exhibited preferential adsorption of nanoparticles

**Fig. 4:**
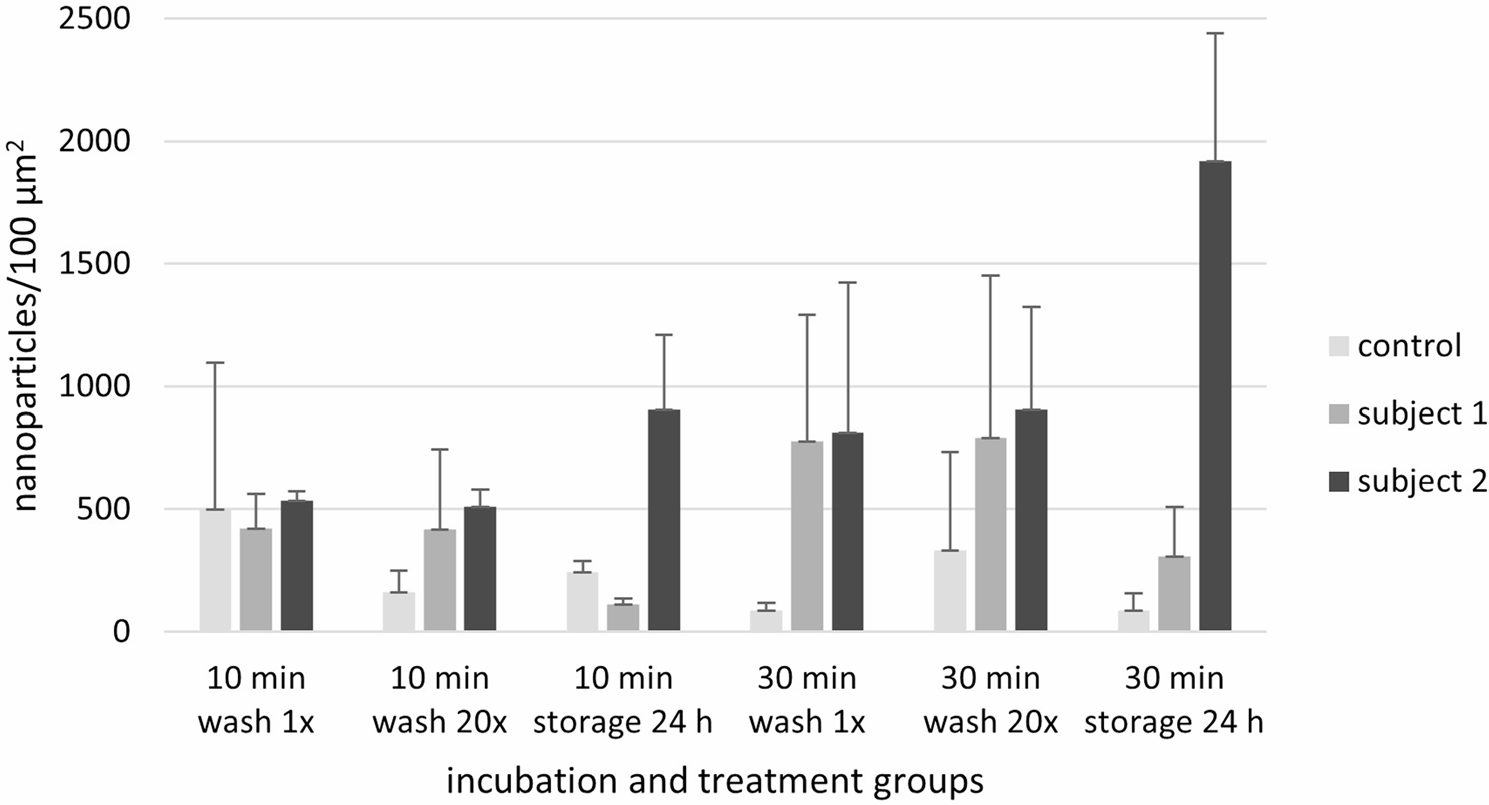
Number of gold nanoparticles quantified using MATLAB from SEM images of enamel specimen (mean ± standard deviation). Specimens were incubated either directly with nanoparticles for 10–30 min (control) or after 24 h of biofilm formation in two subjects. Following incubation, specimens were subjected to either 1 or 20 washes in water or stored in water for 24 h

**Fig. 5:**
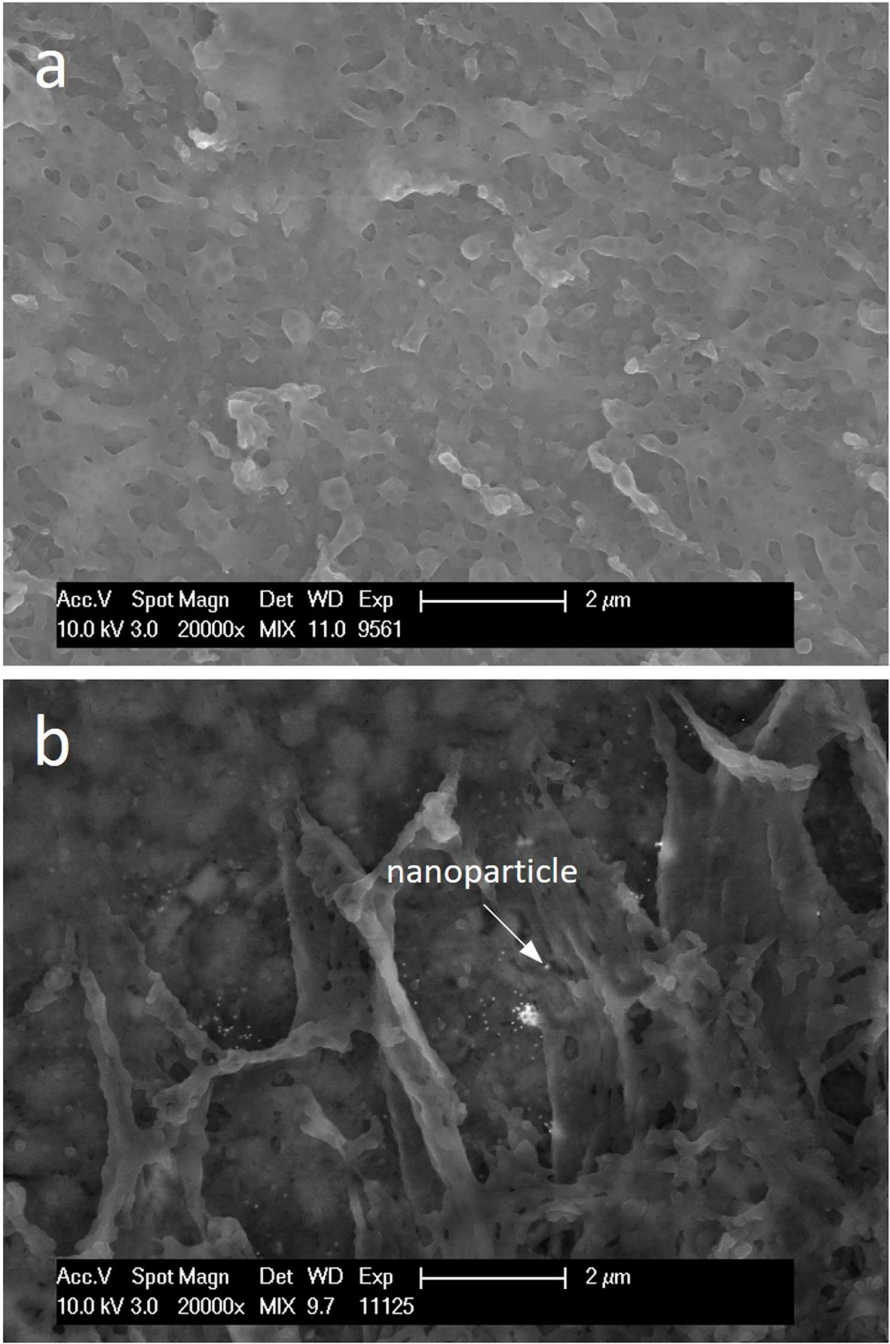
SEM images of the basal layer of pellicles after enamel removal. Biofilm formation was performed in situ, followed by a 10-min incubation with gold nanoparticles and 24 h of water storage

Compared to the control, the enamel was covered by a globular-structured pellicle after 24 h of intraoral exposure. Bacteria adhered to the pellicle, occasionally forming a biofilm, which was more frequently observed in subject 2. Morphologically, the biofilm consisted predominantly of coccoid bacteria. Both the pellicle and biofilm facilitated the adsorption of nanoparticles, which showed no signs of aggregation (Fig. 3). Quantitative analysis revealed that the number of adsorbed nanoparticles increased with exposure time, while the number of water washes had no impact. In subject 2, distinctly more nanoparticles remained on the outer surface when specimens were stored in water instead of being washed. The most notable inter-individual differences were observed after 24 h of water storage (Fig. 4). After the enamel was removed, the basal layer of the pellicle was visualized, which also appeared globularly (Fig. 5). The number of calculated nanoparticles on the outer surface correlated with the observations made on the basal layer. When nanoparticles were homogeneously distributed on the outer surface of the pellicle and biofilm, they appeared on the basal layer in localized areas rather than uniformly, likely confined to thin organic layers such as the pellicle, where gold nanoparticles fall within the SEM interaction volume. However, it is not possible to determine whether the nanoparticles are located on or beneath the pellicle using SEM alone. This distinction was further clarified through TEM.

### TEM analysis

**Fig. 6:**
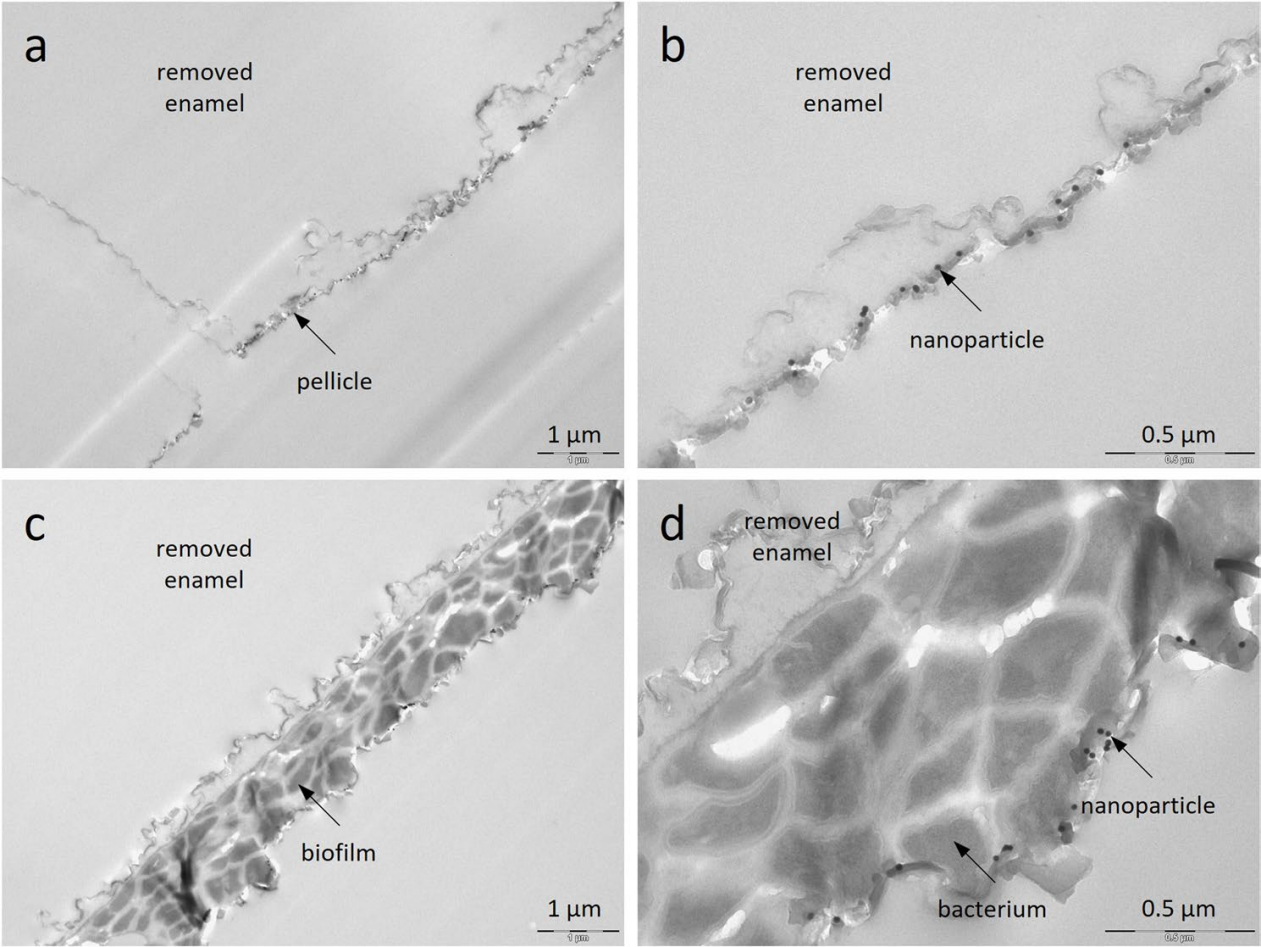
TEM images of enamel specimens exposed intraorally in subject 2, incubated with gold nanoparticles for 30 min, and washed once with water. Enamel was removed prior to sectioning. The cross-sections show the pellicle (**a**, **b**) and biofilm (**c**, **d**) with electron-dense nanoparticles adsorbed onto their outer surfaces

Due to the unconventional sample preparation for TEM, the organic structures are shown less accurately (Fig. 6). The pellicle appears as a thin layer, while the biofilm is compressed, with individually distinguishable bacteria. The gold nanoparticles, visible as electron-dense spheres, are deposited on the outer surfaces of the pellicle and biofilm but are absent in deeper layers.

## Discussion

The nanoparticles were identified in both SEM and TEM due to their gold core. Compared to the control, the nanoparticles preferentially adsorbed onto organic structures, such as the pellicle or biofilm. The nanoparticles adsorbed only to the outer surface and were unable to penetrate the biofilm.

Nanoparticles possess a high specific surface area and, after being transported to the biofilm-fluid interface, can adsorb onto the biofilm outer surface [3]. Although the nanoparticles in the present study had a low surface charge, which could result in poor adsorption due to the lack of electrostatic interactions [11, 12], hydrophobic and van der Waals interactions might enable the adsorption to hydrophobic components of the pellicle or biofilm [11]. The nanoparticles were homogeneously distributed across the biofilm outer surface, consistent with the findings of Li et al. (2015). Their study further demonstrated that hydrophobicity influences the distribution of cationic nanoparticles and determines whether they preferentially localize in the extracellular matrix or bacteria [12]. However, as in the present study, the nanoparticles were applied in vitro, which does not fully reflect the clinical situation. When applied intraorally, salivary proteins could selectively adsorb to the nanoparticles, forming a protein corona. This protein corona may mask or alter the physicochemical properties of the nanoparticles, potentially influencing their adsorption to the pellicle or biofilm [13].

The number of adsorbed nanoparticles remained largely unchanged after 20 water washes, suggesting that weakly bound nanoparticles were removed during the initial wash or that overall desorption is minimal. This aligns with the findings of Sahle-Demessie et al. (2011), who reported that biofilms provide numerous active sites for nanoparticle adsorption; however, their study was conducted using an artificial biofilm [14]. In contrast, Thurnheer et al. (2003) observed a complete loss of dye from a multispecies biofilm after 60 min of washing, indicating the diffusion and desorption of fluorescence-labelled macromolecules due to weak interactions [15]. Additionally, in mature biofilms, the extracellular matrix can degrade, and bacterial detachment may occur, potentially leading to the loss of adsorbed nanoparticles from the biofilm [16, 17]. In the present study, the adsorption kinetics of nanoparticles to pellicles and biofilms appeared to differ, as demonstrated by subjects 1 and 2, who exhibited differing biofilm formation rates. A decrease in the number of nanoparticles was observed in subject 1, whereas an increase was noted in subject 2. Given that nanoparticles adsorb differently to various structures [3], we speculate that, during 24 h of water storage, weakly bound nanoparticles initially desorbed from the pellicle in subject 1. In contrast, in subject 2, initially desorbed nanoparticles may have re-adsorbed from the solution to the biofilm over the same period. However, the low number of subjects in this study does not allow for definitive conclusions.

Although nanoparticles adsorbed to the specimens, they were unable to penetrate the biofilm, even after 30 min of incubation and an additional 24 h of water storage. The ability of nanoparticles to diffuse depends on various characteristics of both the particles and the biofilm. Within the biofilm, diffusion occurs through pores that vary in size and are often tortuous, effectively making the biofilm a size-selective barrier [7, 15]. Moreover, the biofilm, particularly the extracellular matrix, carries a negative charge, which facilitates the adsorption and diffusion of positively charged nanoparticles [17]. The surface properties of bacteria also appear to play a role, as hydrophilic bacterial surfaces enhance the diffusion of negatively charged nanoparticles [18]. While the effective pore size of dental biofilms formed in situ remains unknown, the lack of diffusion observed in the present study may be explained by the size of the nanoparticles and their low-charge surface coating [12]. Since pore size is likely associated with biofilm density, and only one of the two included subjects developed a notably thick biofilm, a comparison of density-related diffusion was not feasible. Additionally, when nanoparticles are applied intraorally, they are likely to increase in size due to the formation of a protein corona [13]. The size of nanoparticles appears to play a primary role compared to their surface charge. In general, the diffusion of macromolecules and nanoparticles decreases as their size increases. Notably, penetration is distinctly reduced for particles larger than approximately 50 nm, mainly due to the size exclusion effect [7, 15, 19, 20].

In the present study, the biofilm was fixed prior to the application of nanoparticles to prevent their diffusion and desorption during the multiple washing steps required for sample preparation in electron microscopy. However, this approach introduces another limitation, as no fixative can optimally preserve all structures of a biofilm. A cross-linking fixative was used to preserve the bacterial morphology, but it may cause the collapse of the extracellular matrix [21, 22]. This collapse could result in changes to the pore size, ultimately impairing diffusion. Additionally, the physicochemical properties of the biofilm might be altered due to the fixative binding to functional groups. The fixed biofilm also differs from a living, dynamic biofilm in its inability to develop resistance. Biofilms can adapt to nanoparticles by increasing the production of the extracellular matrix, which hinders nanoparticle diffusion [11]. Another limitation is the static application of nanoparticles. The interaction of nanoparticles with the biofilm could be enhanced by the high velocity and turbulent flow of a mouth rinsing, as this reduces the external mass transfer resistance [23]. Moreover, the prolonged application times of 10 and 30 min do not reflect clinically relevant conditions; nevertheless, no nanoparticle penetration into the biofilm was observed.

The diffusion of nanoparticles has been investigated in most experimental studies using mono- or multispecies in vitro biofilms. However, extrapolation to intraorally formed biofilms is limited, as these are dynamic, highly individual, and consist of over thousand different species [24]. In the oral cavity, nanoparticles may also influence biofilm formation through interactions with planktonic bacteria and salivary proteins. Additionally, even if nanoparticles cannot diffuse through the biofilm, with or without a protein corona, they may become entrapped within the biofilm after adsorption, subsequent bacterial adhesion, and the formation of the extracellular matrix [16, 25]. Thus, nanoparticles represent a potential strategy for the treatment of biofilm-associated diseases, such as caries. For instance, nanoparticles could act as drug carriers incorporated into the biofilm, where a dysbiotic biofilm with a pH drop could trigger the disintegration of the nanoparticle envelope, releasing antibacterial substances [26].

Regardless of the potential of nanoparticles, their toxicological assessment remains the greatest limitation. Due to the high variability of nanoparticles, toxicity must be evaluated on a case-by-case basis, as the size, shape, and surface chemistry can significantly influence their toxicological profile. Many toxicology studies rely on cell and animal models, which have limited ability to predict human responses and adverse effects, particularly over the long term [27]. Therefore, a critical evaluation of the benefit-cost ratio is essential.

## Conclusion

In conclusion, 20 nm nanoparticles adsorbed to biofilms and pellicles; however, no penetration into the biofilm was observed, highlighting potential barriers related to the biofilm’s structural and physicochemical properties. It is important to note that the biofilms were pre-fixed prior to nanoparticle application, which may have altered key characteristics relevant to these interactions. While the findings suggest possible challenges for the use of nanoparticles in biofilm management, further research is needed to investigate their interaction with dynamic, living biofilms in the oral cavity, along with a comprehensive evaluation of their toxicological profile to ensure safety and efficacy in clinical applications.

## Data Availability

The datasets used and/or analysed during the current study are available from the corresponding author on reasonable request.

## Abbreviations

SEM: Scanning electron microscopy
TEM: Transmission electron microscopy
